# Tuberous sclerosis complex-associated neuropsychiatric disorder (TAND) in a low-resource setting – From seizure to psychosis: A case report

**DOI:** 10.1016/j.amsu.2020.11.084

**Published:** 2020-12-01

**Authors:** Shashwat Pokharel, Pallawi Jyotsana, Robert Singh Maharjan, Rajshree Singh, Kamal Pandit

**Affiliations:** a Kathmandu Medical College and Teaching Hospital, Kathmandu, Nepal; b Nepalese Army Institute of Health Sciences, Kathmandu, Nepal; c Tribhuvan University Teaching Hospital, Institute of Medicine, Kathmandu, Nepal

**Keywords:** Tuberous sclerosis complex (TSC), Psychiatric disorder, Seizure, TAND-checklist, MRI, Nepal

## Abstract

**Introduction:**

Tuberous sclerosis complex (TSC) patients commonly present with neuropsychiatric symptoms – grouped as TSC-associated neuropsychiatric disorder (TAND) - incorporating Autism Spectrum Disorder (ASD) symptoms, intellectual and learning disabilities, psychiatric and behavioral problems. A structured symptomatic assessment known as the TAND-checklist can be useful in reviewing these symptoms systematically and comprehensively.

**Case summary:**

A 21-year-old woman presented with delusions of reference, auditory hallucinations, irritability, restlessness, aggressive behavior, new-onset tremors and rigidity in both upper limbs, and refusal of food and medication intake for 1 week. She has a history of several seizure episodes since 3 years of age which was controlled on oral sodium valproate, carbamazepine, and clobazam. MRI revealed tubers in frontal and insular cortex.Ultrasound of the abdomen showed bilateral renal angiomyolipomas. She was diagnosed with TSC with psychotic symptoms.

**Discussion:**

TSC2 mutations usually present early with epileptic spasms (ES), complex epilepsies, intellectual and cognitive deficits, cardiac rhabdomyomas, and sub-ependymal giant-cell astrocytomas (SEGAs) with high tuber-to-brain proportions (TBP). There is also a remarkable symptom overlap between autism spectrum disorder (ASD) and TSC with behavioral/psychiatric disorders. Social and behavioral problems seen in our patient may be a manifestation of either TSC, ASD, or both. Cost-effectiveness and pragmatism must be considered for TAND-patients in low-resource settings. While it may be theoretically valid to seek genetic testing, TBP-measurement, and mTOR-inhibitor therapy to address TAND-symptoms, they are impractical when compared to TAND-checklist during follow-up.

## Highlights

• Tuberous sclerosis complex (TSC) patients commonly present with neuropsychiatric symptoms – grouped as TSC-associated neuropsychiatric disorder (TAND) - incorporating Autism Spectrum Disorder (ASD) symptoms, intellectual and learning disabilities, psychiatric and behavioral problems.
• Cost-effectiveness and pragmatism must be considered for TAND-patients in low-resource settings. TAND-checklist can be useful in reviewing TAND symptoms systematically and comprehensively.
• Tuber-to-brain proportion (TBP) from imaging studies also help predict cognitive and neuropsychiatric disorders better than only radiologically summing up the number of tubers at a certain age; and TBP is inversely related to age at seizure onset, and cognitive index.

## 1 Introduction

Tuberous sclerosis complex (TSC) is an autosomal-dominant disorder due to mutations in TSC1 and TSC2 genes. In 85% of TSC, 31% have TSC1 mutation (hamartin gene) and 69% have TSC2 mutation (tuberin gene) [1]. Over 90% of diagnosed TSC patients commonly present with neuropsychiatric symptoms – grouped as TSC-associated neuropsychiatric disorder (TAND) - incorporating Autism Spectrum Disorder (ASD) symptoms, intellectual and learning disabilities, psychiatric and behavioral problems [[1], [2], [3]]. TSC2 mutations have worse neuropsychiatric manifestations [1]. However, a structured symptomatic assessment known as the TAND-checklist can be useful in reviewing these symptoms systematically and comprehensively [2,3]. The aim of this Case report is to highlight the usefulness and cost-effectiveness of using a TAND-checklist during follow-up in an actual TAND case in a Nepal.

## 2 Case description

A 21-year-old woman presented with delusions of reference, auditory hallucinations, irritability, restlessness, aggressive behavior, new-onset tremors and rigidity in both upper limbs, and refusal of food and medication intake for 1 week. Until recently, these symptoms were well-controlled on Risperidone. She has a history of several seizure episodes since 3 years of age: the latest of these were two episodes of generalized tonic-clonic seizures (GTCS) seven months prior – now controlled on oral sodium valproate, carbamazepine, and clobazam. Despite her mother not receiving pre-natal care, she had normal growth and development until age 5, after which she developed learning and social difficulties.

On examination, she had ash-leaf spots over her back and angiofibroma over her face. Neurological examination revealed tremors and rigidity in both upper limbs. Her affect was fearful, with delusion of reference and hallucinatory behavior and a poor insight.

### 2.1 Investigations

**Fig. 1:**
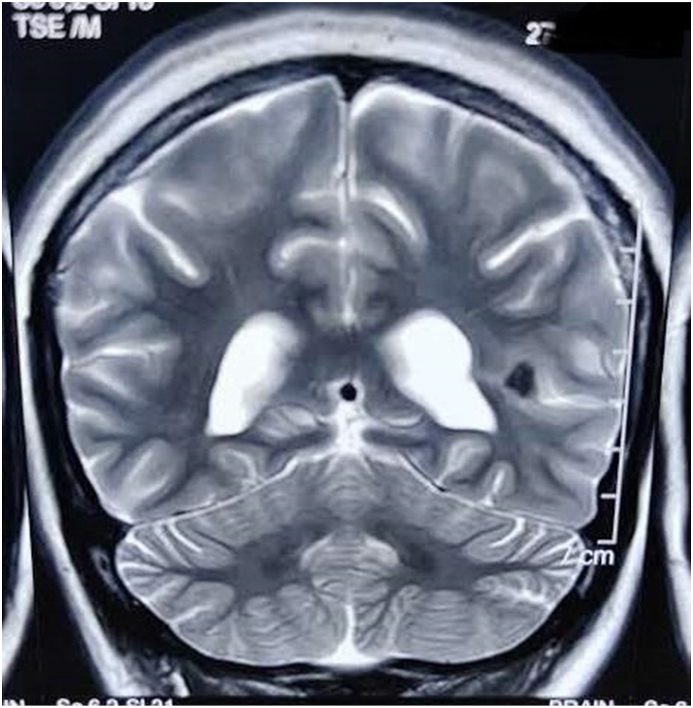
Tubers in frontal and insular cortex.

**Fig. 2:**
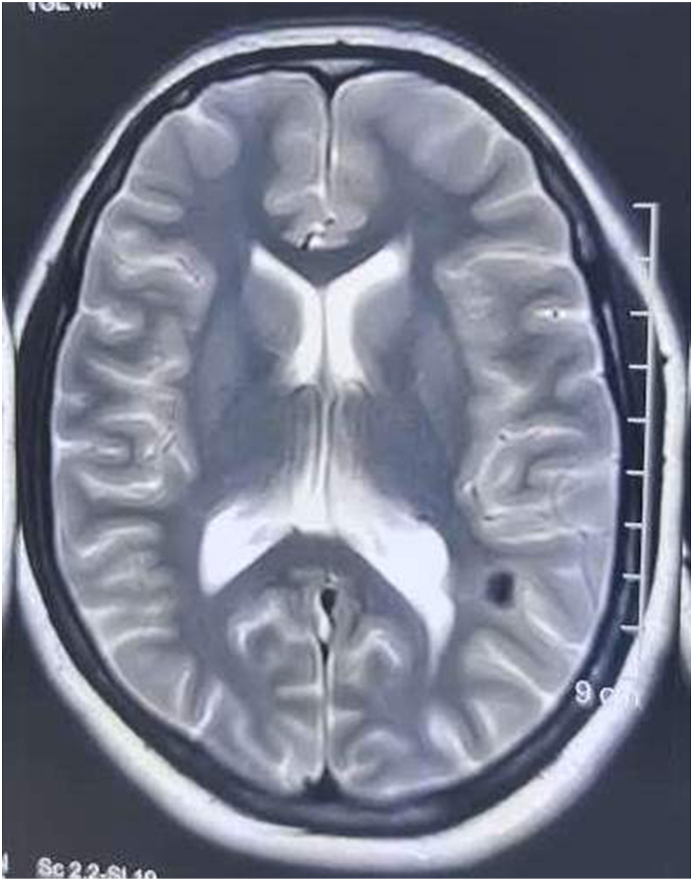
Tubers in frontal and insular cortex.

Routine laboratory investigations were within normal limits. However, Non contrast MRI brain revealed tubers in frontal and insular cortex (Fig. 1, Fig. 2). Ultrasound of the abdomen showed bilateral renal angiomyolipomas. She met the diagnostic criteria for Tuberous Sclerosis Complex (TSC) [Table 1] with psychotic symptoms [4].

**Table 1:** TAND-checklist design.

Item	Level of investigation
Question 1	Primary developmental milestones
Question 2	Level of functioning
Question 3	Behavioral problems
Question 4	Diagnosed psychiatric disorders
Question 5	Intellectual disability
Question 6	Academic performance
Question 7	Neuropsychology
Question 8	Psychosocial functions
Question 9	Parent/caregiver rating of the impact of TAND
Question 10	Priority list for future
Question 11	Additional concerns
Question 12	Physician/Health-care professional rating of the effect of TAND

### 2.2 Treatment, outcomes and follow ups

Treatment was started with trihexyphenidyl for upper extremity tremors and rigidity which were neuroleptic-induced extrapyramidal symptoms.We started monthly fluphenazine injection due to her noncompliance to risperidone. Within days, hallucinations improved, and we discharged her. However, after 6 months, her psychotic symptoms relapsed due to non-compliance. We continued the same regimen after re-admission and subsequent discharge, but this time used the TAND-checklist during each bi-annual follow-up. She currently stays symptom-free after 9 months.

## 3 Discussion

TSC2 mutations usually present early with epileptic spasms (ES), complex epilepsies, intellectual and cognitive deficits, cardiac rhabdomyomas, and sub-ependymal giant-cell astrocytomas (SEGAs) with high tuber-to-brain proportions (TBP) [1]. Cardiac Rhabdomyomas may suggest an early diagnosis of TSC if detected by USG in-utero – possibly missed in this patient due to absent prenatal checkup. Well-baby-clinics could have overlooked ES during infancy in this patient – leading to unwanted epileptic and cognitive outcomes.

There is also a remarkable symptom overlap between autism spectrum disorder (ASD) and TSC with behavioral/psychiatric disorders [5]. Social and behavioral problems seen in our patient may be a manifestation of either TSC, ASD, or both. It would be proper to group our patient into a novel category called TSC-associated neuropsychiatric disorder (TAND). 38–80% of TSC patients have learning disorders [6], and TSC2 mutations tend to have worse TAND-symptoms [1]. A 2015 pilot study of TAND-checklist suggests that we should apply the checklist to all TSC patients who present with any one symptom/disorder falling within the TAND spectrum during regular follow-ups [2,3]. TAND checklist therefore provides a comprehensive evaluation of each symptom-dimension in the form of 12 questions [Table 1] [2]. We used the TAND-checklist on our patient during each follow-up and realized its effectiveness in holistic management of TAND as she has remained symptom-free due to improved medication compliance. The impact/burden on the patient and family was 7/10 and 6/10 as per two consecutive TAND-checklists. It is easy to miss symptoms without the checklist - failure to address them in a systematic and timely manner may lead to treatment delays and worsen cognitive outcomes. From our experience, the TAND-checklist would be even more effective if translated into local language [2,3].

Newer studies show FDA-approved mammalian target of rapamycin (mTOR) inhibitors such as sirolimus and everolimus are effective in reducing SEGA volumes [1]. Surgical resection remains the definitive treatment for smaller SEGAs, but mTOR inhibitors may benefit in complex, unresectable ones [1].^1^ However, it is unsure how pragmatic such modalities may be in resource-poor settings.

Seizures are very common in TSC, usually presenting as easy-to-miss epileptic spasms (ES) during infancy; approximately 83–85% of TSC patients report some form of seizure in their lifetime like in our patient [7,8]. Seizure frequency correlates well with later cognitive disorders in most patients: younger the age at seizure onset, lower the intelligence and cognitive index [9]. Addressing seizures early might improve neuropsychiatric outcomes [1]. While seizures may be refractory in 63% of TSC patients with epilepsy, 37.5% may be seizure-free after treatment [7]. Genetic testing for TSC2 mutations in childhood may also help prepare for better seizure-control with appropriate anti-convulsants and prevent cognitive/intellectual sequelae; however this may not be cost-effective in low resource settings.

Tuber-to-brain proportion (TBP) from imaging studies also help predict cognitive and neuropsychiatric disorders better than only radiologically summing up the number of tubers at a certain age; and TBP is inversely related to age at seizure onset, and cognitive index [9]. Our patient's TBP was not measured.

Cost-effectiveness and pragmatism must be considered for TAND-patients in low-resource settings. While it may be theoretically valid to seek genetic testing, TBP-measurement, and mTOR-inhibitor therapy to address TAND-symptoms, they are impractical when compared to TAND-checklist during follow-up. TAND-checklist in neurological and psychiatric outpatient departments will help physicians deliver a holistic and patient-centered care to TSC patients at no extra cost.

## Ethical approval

This is a case report. This is not a research study so ethical approval is not required.

## Source of funding

There is no any source of funding for this case report.

## Author contribution

1. Shashwat Pokharel took relevant history, clinical examination, collected relevant investigations and follow up assessment of the patient.He wrote and edited the report.

2. Pallawi Jyotsana, Robert Singh, Rajshree Singh, Kamal Pandit also wrote the report and revised it with relevant references.

## Research registration number

1. Name of the registry: Not Applicable.

2. Unique Identifying number or registration ID:

3. Hyperlink to your specific registration (must be publicly accessible and will be checked):

## Guarantor

Shashwat Pokharel.He is the first author and corresponding author for this case report.

## Consent

Written informed consent was obtained from the patient for publication of this case report and accompanying images. A copy of the written consent is available for review by the Editor-in-Chief of this journal on request.

## Provenance and peer review

Not commissioned, externally peer reviewed.

## Declaration of competing interest

There is no any conflicts of interest.
